# "The Hospital" Nursing Mirror

**Published:** 1897-04-24

**Authors:** 


### "The Hospital" Nursing Mirror.

Being the Nursing Section of "The Hospital."

[Contributions for this Section of "The Hospital" should be addressed to the Editor, The Hospital, 28 & 29, Southampton Street, Strand, London, W.C., and should have the word "Nursing" plainly written in left-hand top corner of the envelope.]

### News from the Nursing World.

### A WARNING TO NURSES.

A correspondent writes to us to tell of experiences of her own, which may, she thinks, warn others who intend taking up nursing as a profession against similar pitfalls. Desiring to train, she sought advice from a nurse who "undertook to train probationers," and to whom she paid a premium, binding herself for two and a half years, only to realise long before the end of that time that she had been caught "in the net of private enterprise," to her own disadvantage, and to the benefit of the receiver of the premium. "I would ask you," says our correspondent, "to warn all would-be nurses to avoid entering the profession through any 'loophole' that may offer itself, and not to take the step until they have full knowledge of the best ways and means from some reliable authority."

### LEGAL PROCEEDINGS.

An action to recover £50 damages for alleged wrongful dismissal from the Belfast Maternity Hospital was recently brought by Grace Tierney, a pupil at the hospital against the committee. The case came before the Recorder of Belfast and excited a good deal of local interest. Certain charges were made against Nurse Tierney (who, it was stated, held a certificate of training from the Adelaide Hospital, Dublin), of which she denied the truth. In summing up, the Recorder said that the plaintiff having been "hired as a pupil" was subject to the rules of the hospital, and that therefore the committee had power to dismiss her if she proved unsuitable. Even if a rule had not existed the committee were entitled to exercise their discretion, and under these circumstances the action was not sustainable.

### MIDDLESEX HOSPITAL CHAPEL.

To anyone with a couple of thousand pounds to spare, who wishes to find an excellent object on which to spend his money, we would commend a visit to the Middlesex Hospital and its beautiful little chapel. Such a visitor would surely feel an irresistible inclination to complete the work so well begun and make the chapel the most perfect of its kind in all London. The decoration is after the designs of Mr. Pearson, and the varied effects of the exquisite marble and mosaic work compel unstinted admiration; but alas! there has not been enough money given to complete this expensive treatment, and a portion of the walls and of the roof remains in the rough, awaiting a gift from some generous friend to finish it. Three stained glass windows have been given and another promised, but more than anything the unfinished walls appeal to all beholders for the funds which would enable the beautification of this little "House of God" to be worthily completed. Will not some rich folk go and see for themselves what is wanted, and having seen give their alms for this purpose?

### DOLLS FOR DISTRICT NURSES.

Miss Thomson, the secretary of the Victoria Commemoration Club, has, on behalf of The Hospital, undertaken the charge of a large number of dolls dressed in the uniform of nurses of various hospitals in London and the country, and has consented to distribute them amongst any district nurses or others who would care to have them to give away to small patients. The dolls are not new, but the clothing is good and neatly made, and, for the most part, of washing materials; and we are glad to accept Miss Thomson's offer to find recipients for them. Nurses can receive the dolls from Miss Thomson at the Club, 29, Southampton Street, Strand, W.C.

### TRAINING AT SWANSEA HOSPITAL.

New arrangements have been entered into regarding the training of probationers for the Nursing Institute at the Swansea Hospital. The term of training has been fixed at two years, a fee of £10 for each nurse being paid by the institute to the hospital. Two probationers will be taken each year. They will be boarded by the hospital, and they will be under the absolute control of the matron. Certificates will be granted at the end of the two years.

### NURSING IN CORNWALL.

Lord St. Levan presided over a meeting recently held at Penzance to discuss the County Nursing Institute which it is proposed to establish for Cornwall. Lord St. Levan explained that the institute would interfere with no existing organisation, but would help those which elected to become affiliated with it. A motion to constitute the Hundred of Penwith a nursing district in connexion with the Institute was carried, and a committee was appointed to set about the work of collecting subscriptions and donations in support of the scheme. The project has met with universal approval. The committee will presently make a report to an adjourned meeting.

### LIVERPOOL NURSES' INSTITUTION.

At the recent annual meeting of this institution it was stated that the nurses' earnings for the past year exceeded those for the year before by £200, and there was a balance in hand at the close of the twelve months of £239. The chairman, Dr. E. R. Bickersteth, in moving the adoption of the report, spoke of the "satisfactory balance," and said that it was decided to give out of the surplus a sum of £100 towards the building fund of the new consumption hospital, and to place £50 at the disposal of the Ladies' Committee to spend as they might think fit in commemorating the Queen's Diamond Jubilee. A writer to the *Liverpool Post* has drawn attention to this matter, and as "one of many who have . . . benefited by the skilful services of nurses from this most valuable institution," asks "whether it is right or just to hand over money which has been earned by the nurses to any charity, however good.

until the future comfort of those who by their faithful and untiring efforts make this 'satisfactory balance' has been secured." Had this been so the writer's criticism would be well deserved, but we understand that the real facts of the case are as follows: So far from the nurses' earnings being given away, the committee spend upon them each year some £100 or £200 more than they earn, and the amount it is now proposed to give to other charities comes from the invested funds of the institution, money given in donations and legacies in its early years when it was the only nursing home in Liverpool. The hon. secretary informs us that the £50 to the Ladies' Jubilee Committee was voted at the annual meeting in consideration of the wish of the committee to provide some special treat for the nurses this summer.

### LEICESTER INSTITUTION OF TRAINED NURSES.

At the annual meeting of the Leicester Institution of Trained Nurses a favourable report was presented by the committee, the financial position showing an improvement on that for the previous year. We are glad to note that the committee contemplate, should the present year prove as successful as the last, giving their private nurses "a personal interest, beyond their stipends," in their work. The district nursing branch, which from the report appears to be, as it ought, entirely distinct in maintenance and management from the private nursing branch of the institution, needs increased subscriptions if its work is to be kept up as it should be. There are eleven nurses now working in Leicester, and their helpfulness meets with general recognition. Nurse Barnes and Nurse Marsland have left the staff, and their places have been taken by Nurses Baker and Wallace.

### TAVISTOCK GUARDIANS AGAIN.

It will be remembered that the Local Government Board and the Tavistock Guardians have had a recent difference of opinion over the action of the latter in appointing an untrained girl as "assistant nurse" at the Union infirmary, there being no trained nurse in the establishment at all. Six Guardians petitioned the Local Government Board not to sanction the appointment, that body responding by a request to the Board to "reconsider" the matter. At the last Board meeting, a resolution was passed by these enlightened Guardians to the effect that "the majority should adhere to their resolution, and send a statement to the Local Government Board justifying the action they had taken." It was moved as an amendment that consent be asked to the continuance of the present arrangement for six months, and that a more efficient nurse be then appointed. Twelve voted for the amendment, and 23 for the original motion. It is to be hoped the L.G.B. will insist upon the Guardians' appointment of a proper nurse. There are 40 to 60 unfortunate sick people now left to the sole care of one untrained "nurse" and an assistant declared by both this "nurse" and the medical officer to be entirely unfit for her position. It is a shameful state of things.

### GUY'S HOSPITAL NEW NURSES' HOME.

Guy's Hospital has received a munificent gift of £1,000 from the Baroness de Hirsch towards the erection of the proposed Nurses' Home.

### WORKHOUSE NURSING.

A recently-issued Parliamentary paper, dealing with the character of the nursing in workhouses and poor-law infirmaries in England and Wales, gives statistics showing the relative proportion of trained nurses to pauper attendants throughout the country last year. The return shows that in the 30 London unions last June there were 1,514 "paid officers acting as nurses," 848 of whom were trained, while there were 349 pauper inmates employed as "assistant nurses." The pauper help varied in the various unions from 46 in Green wich, where 66 paid nurses were employed, to only two in the West Ham Union, where the paid nurses numbered 36. In the divisions outside London there were 2,204 paid officers acting as nurses, of whom 1,113 had received training before appointment, and 3,094 pauper inmates assisting in the care of the sick. Liverpool, Birmingham, and Sheffield lead the way in reform in this matter, and in these unions no pauper help is employed at all.

### THE SARAH ACLAND HOME FOR NURSES.

It is announced that the Prince of Wales will open the new buildings of the Sarah Acland Home for Nurses at Oxford on May 12th. The Prince is to be the guest of the Dean and Mrs. Paget, and earlier in the day will inspect the Queen's Own Oxfordshire Hussars and open the new municipal buildings.

### ENGLISH NURSES FOR THE EAST.

Four English nurses left England on Good Friday for Athens. They were Miss Isabel Carter, Miss Ellen Pillott, Miss Katherine Stollard, and Miss Alice Davies. These nurses have been selected, at the request of the Princess of Wales, for the Crown Princess of Greece, who, we understand, will defray all the attendant expenses of the project, which has nothing whatever to do with Mrs. Ormiston Chant's scheme. The Crown Princess of Greece arrived at Larissa last week, accompanied by ten English ladies of the Red Cross Society, in order to establish an ambulance hospital.

### NURSING IN SOUTH AFRICA.

A letter in a recent issue of *South Africa* appeals to South Africans in London to lend their help towards the nursing institute which it is proposed to establish at Cape Town in commemoration of the present year. "The Victoria Nurses' Institute" is to be founded on co-operative lines, and it is confidently expected will very quickly prove self-supporting. Subscriptions are asked for the first three years, so that the institute may be well equipped and efficiently started on its career. Seeing that South Africa is doubtless "the country of the future," and that the demand for well-trained nurses must be an ever-increasing one, such an institute should, properly managed, be eminently successful. Donations and subscriptions may be sent to the Editor of *South Africa*.

### SHORT ITEMS.

Miss E. O. Wildman has returned from Gibraltar, and is now at St. Thomas's Home; she has not yet recovered from the very serious accident she sustained at sea in December last. Miss Watts is acting as deputy superintendent for her at the Station Hospital, Secunderabad.

### Pharmacy and Dispensing for Nurses.

By C. J. S Thompson.

### VIII.—METHODS OF ADMINISTERING MEDICINES—MIXTURES.

The various methods employed for the administration of medicine may be divided into two classes, viz.: (1) Medicines for internal use, (2) medicines for external application. The former include mixtures, draughts, drops, emulsions, pills, powders, cachets, and tablets. Suppositories and pessaries are administered per rectum. The latter consist of liniments, lotions, ointments, pastes, plasters, and blisters.

On receiving a prescription to compound, the dispenser's first duty is to carefully read it through in order to consider the best method of preparing it. If intended for internal use, the dose of each ingredient should be noted and the frequency of its administration. It should then be noticed if any of the ingredients ordered are likely to be incompatible when brought together, and, if so, precautions must be taken to prevent such reaction as far as possible. The course of preparation adopted should be that which will best preserve the properties of the drugs used, and which will also exactly carry out the intentions of the prescriber.

### Mixtures.

The mixture is the name given to a liquid medicine for administration by the mouth, generally in doses ranging from one drachm to two ounces, according to the directions of the prescriber. The quantities prescribed vary from one to twenty ounces. The mixtures usually ordered are two, four, six, eight, ten, or twelve ounces. They may consist of solid substances suspended or dissolved in water or in a watery medium, consisting of an infusion or decoction combined with other preparations, or they may take the form of a simple mixture of tinctures, spirits, and syrups, diluted with water.

When dispensing a prescription it is not necessary to mix the ingredients in the order in which they are written. Where solid bodies are ordered they should be dealt with first, and, if soluble, they should be at once dissolved in a portion of the water or other liquid with which the mixture is to be made up. This is done by placing them together in a bottle and shaking well till dissolved. When a mixture is simply composed of liquids the tinctures or other preparations should first be mixed together, and water then added to make up the quantity ordered. If, as we have previously stated, any of the ingredients are likely to produce a chemical reaction when mixed together, and such is evidently not the intention of the prescriber, endeavour must be made to prevent it as far as possible. Thus, if an acid be brought into contact with an alkaline body, we shall have a reaction that will probably burst the bottle. Such antagonistic bodies should be well diluted before being mixed together. Hydrocyanic acid and other volatile liquids, when ordered, should always be added to the mixture last of all, and the bottle corked immediately afterwards.

When making a mixture some dispensers prefer to mix the various ingredients in a glass measure of suitable capacity, and then, when it is completed, transfer the whole to the bottle, but in most cases the best plan is to mix the various articles in the bottle, then adding the water, transfer the whole to a measure to confirm the quantity is correct.

When insoluble bodies such as the carbonates of bismuth or magnesia are included in a mixture, they should be placed in a mortar and well triturated with a portion of the water till thoroughly diffused throughout the liquid. Mucilage is usually added to mixtures containing salts of this kind in order to suspend them. To all bottles containing mixtures in which there is any insoluble matter, a "shake the bottle" label should be affixed. Some medicinal agents, such as Butyl-chloral hydrate, codeine, &c., are much more soluble in spirit than water. When drugs of this kind are met with in a mixture, advantage should be taken of any tincture or spirit included in the prescription to act as a solvent before adding the water.

In some cases, as, for instance, when bulky crystals such as sulphate or phosphate of soda, or sulphate of magnesia in quantity are prescribed, which take a long time to dissolve in the cold, hot water may be used.

To illustrate the compounding of a prescription for a mixture, we will describe exactly how the following should be dispensed: ℞. Potass bicarb, ʒii.; potass bromid, ʒi.; tinct. calumb., ℥ss.; spts. chlorof., ʒi.; syr. aurant., ℥i.; aquæ ad ℥viii. Misce Fiat mist.

In this prescription we have two solid and three liquid ingredients to be made into a mixture with water. It will be well to note here, that when the word "aquæ" is followed by "ad" or "adde," sufficient water is to be added to the other ingredients to make up the mixture to the given quantity. When no directions are given "to add," the exact quantity of water ordered is to be used. To proceed with the mixture, an eight-ounce bottle should be half filled with water, and the bromide of potassium and bicarbonate of sodium having been carefully weighed and placed on a piece of paper, may then be introduced into the bottle. As both these salts are readily soluble in water, if the bottle is shaken briskly for a minute or two solution will soon be effected. When this is done, measure the requisite quantities of tincture of calumba, spirit of chloroform, and syrup of orange, and add to the solution in the bottle. Shake again to mix well, and add sufficient water to fill the bottle. Before corking see that the mixture measures exactly eight ounces by transferring it to a glass measure; also examine it carefully for the presence of any foreign matter or dirt that may have found its way in. If such be present, strain the liquid back into the bottle through a little fine tow or absorbent wool, then cork, shake well for a few minutes, and the mixture is ready for labelling,

But it must not be supposed that every mixture is as easy to prepare as the one we have just described, and we will now consider some of the difficulties the dispenser is likely to meet with, and the best methods of dealing with them. First as to solubility. The student will find it a good plan to commit to memory the solubility of the ordinary solid substances used in medicine. If the quantity of a solid ordered in a mixture is more than the water will dissolve, the undissolved portion should not be strained out, but directions to shake the bottle should be affixed. Hot water will be found to aid solution in many cases. Thus gallic acid is soluble only 1 in 100 of cold water, and in boiling water 1 in 3, but it rapidly re-crystallises out on cooling. This acid should be rubbed down in a mortar as fine as possible, cold water being added gradually. When ordered together with citrate of potassium this is unnecessary, as it dissolves readily. The frothing caused on shaking up citrate of iron and quinine will speedily subside on the addition of a few drops of rectified spirit. It is necessary when dispensing the potassio-tartrate and the pyrophosphate of iron to rub them down in a mortar with hot water to effect solution. Tincture of perchloride of iron and mucilage of acacia when mixed together form an unmanageable jelly, but if the tincture be well diluted with water before the mucilage is added, this can be prevented. Tinctures containing resinous matter, such as guaiacum and Indian hemp, on dilution with water, will precipitate their resin. This may be avoided if the tincture be triturated with a small quantity of mucilage of tragacanth or acacia and the water then added gradually.

### Post-Graduate Clinics for Nurses.

By a Trained Nurse.

### X.—THE HEALTH OF THE PRIVATE NURSE.

I once heard a private nurse remark pathetically, "I do wish some Training School would institute a Professor of Deportment for the instruction of those of its nurses preparing for private practice. It seems so hard to qualify up to the standard and to emerge unscathed from the strong searchlight of criticism thrown upon us. And everybody seems ready to blame rather than to praise the nurse, even though she is trying her very best."

This is a critical age, and the present generation is hard to please. But, considering how far the critical faculty is developed in this our day and making allowance for the peevishness and want of enthusiasm consequent on illness, it speaks very highly for their characters and dispositions that nurses stand so high in the public estimation. The patients in our Institutions have the utmost regard for the "hospital nurse." Persons of a better class judge the nurse chiefly by those they meet in private life, for of the hospital worker they know very little. Although classed together the two types are, in reality, very dissimilar, in spite of the fact that under existing regulations their training is exactly the same. This fact constitutes the great defect in the present methods of Training Schools. But I wait hopefully for a speedy remedy in this defect, and am looking for a progressive Matron who will evolve special classes and courses of study and practice to meet the needs of the budding private nurse. The initial step has already been taken at the London Hospital, and the scheme will probably enlarge and extend till a special, differentiated training is grafted on to the "last year in hospital." In her last year of training the woman who elects to be a "district nurse" should be specialised for her distinctive duties. The "private nurse," too, needs much preparation for her prospective work—as does the nurse qualifying for work abroad. At present all go through the same mill, all are levelled so far as possible to the same grade and pattern, so that on emergence from the school to tread widely different paths, it is not surprising if these various nurses discover that their ways are hard to find, and their road beset with many difficulties.

The nurse intending to spend her life in hospital has no "unknown" terrors to face. From the day she enters her first ward, the newest and most guileless of probationers, she easily and imperceptibly imbibes the traditions and customs pertaining to hospital life. She is taught how to address her superior officers—even as the raw recruit is drilled to give his salute according to the code of military discipline—she learns exactly how her relations with the medical staff are bounded. In short, she becomes familiar with the whole duty of a nurse as laid down in the catechism of her Training School. She is helped to carry out her work by the daily "limitations" necessary in hospital; its rules and precepts are a stay and a prop. The private nurse has no such stand-by. She herself when she goes into a private sick-room must originate and frame the regulations and routine. For no two private cases are alike. The sex, age, temperament, disease, and mental condition of the patient all enter into the problem, and each one of these factors is capable of upsetting all her favourite and approved plans of arranging the routine of the sick-room. Many patients have complained to me that "their nurses were so serious and depressing." On the other hand, I have known a bright, cheerful nurse exchanged for one more subdued because, said her patient, "She is always smiling, and it annoys me." Some persons find their nurses too young, others object to "having an elderly woman about," they like "young, bright companionship." Altogether, taking into account the excessive "faddiness" of the age in which we live, I repeat that the regard in which the trained nurse is held is a wonderful tribute to her many excellences.

So far as health and physique are concerned, the private nurse needs a much larger store to draw upon than does her sister engaged in hospital work. Many a delicate woman is enabled to endure constant hard work as a staff nurse or Sister, just because of the regularity of the life. She knows exactly how many hours she has on and off duty. She can apportion her leisure between rest and recreation, lying down when she needs complete repose, going out when she has an instinct for a "freshening blow in the open air." This living by rule in hospital is just that which enables the woman of inferior physique and constitution to spend her life in doing laborious work. The same woman subject to the irregular conditions of private nursing would probably break down in three months. Many persons fail to realise that, however, agreeable variety of life and changes of abode may be, there is always a certain wear and tear of health concerned in going from one case to another. The nurse on her way to a new patient may possibly enjoy the bustle of starting off in her cab. She may have, too, an interesting railway journey and enjoy "fresh fields and pastures new." But underlying her pleasant impressions there is an unconscious strain, an anxiety as to what kind of household she is going to, whether the case will go off well, how she will please her patient, his doctor and his friends. Arriving tired after a long journey, she has frequently to go on duty for several hours, and, though weary, must rally her physical and mental powers to make a good "first impression." It will be a few days at least before she feels really settled and at home. And, perhaps, by this time she may be no longer needed, and will once more start "on her travels" with its accompanying wear and tear and mental worry. All these factors, combined with the irregularity in her rest and recreation attendant on private nursing, makes it very important that the private nurse should start with a good constitution and know how to keep it. And this point is one on which nurses are often careless. Perhaps because their work bears so much upon hygiene and precautionary detail it becomes somewhat of a relief to throw off such considerations in "off times." Some day I hope to write a long and exhaustive treatise on "How to keep in health though a nurse!" For on the nurse's good condition depends not only her own welfare and happiness, but her constitution in the great majority of cases represents her living, the capital on which she must subsist for many years. But none the less is the average nurse a constant votary at the shrine of the teapot! In vain and multiplied tea-drinkings do nurses show their affection and regard for one another. The night nurse returning from her morning walk, tired and needing sleep, finds sundry cups of tea awaiting her, and friendly company in which to drink it. After a "long" or half-day's recreation, the teapot, steaming and fragrant, stands tempting her to stimulation when sleep should await her. And so on to the end of the chapter—but not the end of the tea-pots, for these never reach finality. The private nurse does not, as a rule, find so many opportunities for tea, unless of malice aforethought, and with original sin strong within her, she takes her own "tea set," caddy, and kettle in her box! The "tea habit" grows and increases day by day in the nursing community, and it is undoubtedly productive of the "nerves" and indigestion common in nurses, as in all other classes of women-workers. But it is almost as useless to preach on the "habit" of tea as to indulge in diatribes against the "habit" of a small waist—the means to which should find no place in the private nurse's trunk!

### Everybody's Opinion.

[Correspondence on all subjects is invited, but we cannot in any way be responsible for the opinions expressed by our correspondents. No communication can be entertained if the name and address of the correspondent is not given, or unless one side of the paper only is written on.]

### THE ABUSE OF UNIFORM.

Nurse H. writes: Has it occurred to anyone that the best and simplest way to protect the uniform of nurses from being travestied would be for every hospital, infirmary, and nursing association to register their own particular uniform (if such a thing could be done), and so make it impossible for it to be worn by any but *bonâ fide* nurses?

### THE "UNIFORM" QUESTION.

"A. T. N." writes: With reference to the uniform question I wish to know if it is a breach of etiquette for a nurse to appear at a social gathering, &c. (or where evening dress is worn) in a plain black or blue dress with collar and cuffs? I find to wear uniform always is the easiest and cheapest plan: cycling, boating, &c., excepted. Why should it be required to provide evening dresses for the few occasions when we should wear them, causing so much unnecessary expense? We are always working among God's needy children, who constantly require our pecuniary aid. Is it not more Christlike to give all we can spare to the suffering poor? Jesus Himself said, "Inasmuch as ye did unto one of the least of these My brethren ye did it unto Me."

### ALMSHOUSES FOR NURSES.

"An Old Nurse" writes: I beg to say that the letter from "A Trained Nurse" in The Hospital of March 27th, and that of "Nurse E" of April 10th, on "Almshouses for Nurses," I read with much interest. Some such thought has often occurred to me, but as so much has been and is being done for nurses, I have not liked to mention it. I think it likely that sooner or later some kind of home will be established for nurses, as there is much difficulty about lodgings. People having a decent little house, and wishing to let one or a couple of rooms, wish to get more out of it than nurses' means will allow. Of course those nurses who are having a pretty good salary can afford to pay 15s. or 20s. for a few weeks for rest and holiday. Should these homes be established, I would like them to be initiated by friends connected with the R.N.P. and the J.S.M. Fund. There might be a home at Bournemouth and another somewhere inland to suit different constitutions and states of health. One in London, as some old nurses, though dearly loving the beauties of sea and country, would not like to wholly settle so far from Pension Fund friends and others. I would not have the homes called almshouses, I would be vain enough to have them called the Royal Homes for Nurses, if our beloved President would patronise them, and each home might be named after friends. Each nurse to have a bed-room to her ownself, and if she had the means each to furnish it. There should be a few simple rules to be kept, for the comfort of all concerned. I do not think any nurse could expect to be in a nice home and to pay less than 8s. per week, this to include everything except laundry. Others to pay 10s., or more, according to their means, besides helping to do a little for the house in which she makes her home. They should not be used as hospitals. I think candidates should belong to the Pension Fund, old and retired first on the list, and not anyone helpless to begin with. When the homes were not full paying nurse visitors might be taken. I fancy that if one or two houses could be started they would be self-paying almost directly.

"A Matron," who writes to us on this subject, has omitted to enclose her name and address for the Editor's information. If she will kindly send this we shall be pleased to insert her letter.

### "QUEEN'S NURSES."

"Rural Queen's Nurse" writes: Reading the Rev. A. L. B. Peile's speech in last week's Hospital, I was surprised to see the low esteem in which rural Queen's nurses appear to be held by the London branch. Why should the latter feel in any way disparaged by being attached to the rural branch? Are not rural nurses Queen's nurses? Nurses who were not trained by the institute are generally three years' certificated nurses, besides holding their L.O.S. and district training, and are as well educated and ladies by birth as any that are working in London. We are doing just the same, and perhaps more, work in the country than they are doing in London; why, then, are we considered a different class of nurse?

[Mr. Peile was not alluding to rural *Queen's* nurses, who, of course, are on precisely the same level as their sisters who work in London. He was speaking of the class of "cottage" or "rural nurse," whose training may have been only six months or less. Associations for establishing this class of "nurse" in country districts have recently sprung up all over the country, and under certain conditions the Queen's Jubilee Institute has undertaken to recognise and inspect the work done. These partially-trained women, however, are not in any way "Queen's nurses."—Ed. *T. H.*]

### HOW TO BECOME A DISPENSER.

Referring to our article in the Mirror for April 3rd under the above heading, Dr. Griffith writes from the Hospital for Women and Children, 9, Lupus Street, that dispensing is taught at this institution.

### Where to Go.

The Victoria Commemoration Club, The Hospital Building, 29, Southampton Street, Strand, W.C.—Miss M. Cummings (Associate of the National Health Society) will deliver a course of post-graduate lectures on "Domestic and Personal Hygiene" on Wednesday, May 5th, and the following Wednesdays at 3 p.m. This course of lectures will be free to nurses, and is specially intended for those engaged in private and district work. The following is the Syllabus: Lecture I. The House—Situation and Soil; Construction and Arrangement; Warming and Ventilation; Water Supply and Drainage. Lecture II. Diet—Varieties of Food; Composition of Body; Waste and Repair; The Use and Abuse of Stimulants. Lecture III. Dress—Object of Clothes; Dress Materials; Details of Garments; The Corset: its special dangers. Lecture IV. Occupation—The Laws of Health; Life in Town and Country; The Influence of Work on Health; Exercise. Lecture V. Health in Infancy and Childhood—The Care of Infants and Children; Education: moral, mental, and physical. Lecture VI. Disorders of Health—How to prevent them; Infectious Diseases and Disinfection Nuisances; Hints on Sanitary Laws.

### Presentations.

Miss Clark, who is resigning her position as matron of the Borough Fever Hospital, Runworth, near Bolton, to take the post of Lady Superintendent at the Grafton House Nurses' Institution, has been presented by her nurses, as a token of their goodwill and esteem, with a handsome reading lamp. In Miss Clark the Borough Fever Hospital have had a devoted and energetic worker.

The matron of the Corporation Hospital, Bootle, Liverpool, had a very happy ceremony to perform on the evening of April 13th. Miss Alice Brown, the sister in charge of typhoid wards, was presented with a china afternoon tea-set, tray combined, in amber and gilt, and brass cake-stand, from the matron and nursing staff. Sister Alice takes with her the esteem of all, and every good wish for her future success in her new work as lady superintendent of a District Nurses' Home in Wolverhampton.

### Appointments.

MATRONS.

FOSTER GREEN HOSPITAL FOR CONSUMPTION, BELFAST.—Miss A. McBryde Broun has been appointed Matron of the above institution, which is about to be opened. Miss Broun was trained at the Children's Infirmary, Liverpool, and King's College Hospital. She later held the appointments of ward sister at the Royal Infirmary, Bristol, and at the Leicester Infirmary; matron of the Home for Incurables, Winchester; and assistant matron at the Royal Hants County Hospital, Winchester.

THE JENNY LIND INFIRMARY, NORWICH.—Miss L. Maude Neville has been appointed Matron at the above institution. Miss Neville was trained at the Nottingham Children's Hospital, and has been charge nurse at Guy's Hospital, ward sister at Pendlebury Children's Hospital, matron at the Nottingham Children's Convalescent Home, and temporary matron at Hammerwich Cottage Hospital.

COVENTRY AND WARWICK HOSPITAL.—Miss Lucy Rae has been appointed Lady Superintendent of this institution. Miss Rae received her training at Charing Cross Hospital, where she afterwards held the post of sister in charge of the out-patient department. She was subsequently appointed assistant lady superintendent of the Nurses' Training School, Liverpool.

ISOLATION HOSPITAL, CHEAM.—Miss Margaret Mannering has been appointed Superintendent and Nurse at this institution. She was trained at the South-Eastern Fever Hospital, and has held appointments as nurse at Milton Hospital, Portsmouth; at the City Hospital, Sheffield; and at Hartlepool Hospital, Hartlepool.

ST. GEORGE'S UNION INFIRMARY, FULHAM. — Miss Margaret Hampson has been appointed Matron of St. George's Union Infirmary. She was trained at St. Thomas's Hospital and the Marylebone Infirmary. She acted as assistant matron at the Cork Street Fever Hospital, Dublin, and later as lady superintendent at the same institution.

CORK STREET FEVER HOSPITAL, DUBLIN.—Miss Rae has been appointed Matron at this hospital, where up to the present she has filled the post of assistant matron.

CONVALESCENT HOME FOR WOMEN AND CHILDREN, NEW BRIGHTON.—Miss E. Hallam has been appointed Lady Superintendent of the above institution. Miss Hallam was trained at the Northern Hospital, Liverpool, where she lately held the post of sister.

THOMAS KNIGHT MEMORIAL HOSPITAL, BLYTH.—Miss E. Morgan has been appointed Nurse Matron of this institution. She was trained and later held the post of charge nurse at the Royal Infirmary, Newcastle-on-Tyne.

### Minor Appointments.

CHELSEA HOSPITAL FOR WOMEN.—Miss Emily Boyer, who was trained at Cardiff Infirmary, and has held the posts of ward sister at St. Mary Abbots, Kensington, and nurse at the Samaritan Free Hospital, Marylebone Road, has been appointed Charge Nurse at this hospital.

ASTON UNION INFIRMARY, BIRMINGHAM.—Miss Marion Glynn has been appointed Charge Nurse at this institution, where she also received her training.

### Death in our Ranks.

WE regret to learn that Miss Agnes McCaw, sister in charge of the children's wards at the Royal Albert Infirmary, Wigan, died on March 26th of acute peritonitis after a week's illness. Miss McCaw had been sister for about six months, and she served the little ones committed to her care with the truest devotion. Her bright and amiable disposition made her a favourite with everybody in the infirmary, and her sudden death at the early age of 28 has come as a great sorrow to those who knew her there.

### Notes and Queries.

The contents of the Editor's Letter-box have now reached such unwieldy proportions that it has become necessary to establish a hard and fast rule regarding Answers to Correspondents. In future, all questions requiring replies will continue to be answered in this column without any fee. If an answer is required by letter, a fee of half-a-crown must be enclosed with the note containing the enquiry. We are always pleased to help our numerous correspondents to the fullest extent, and we can trust them to sympathise in the overwhelming amount of writing which makes the new rules a necessity. Every communication must be accompanied by the writer's name and address, otherwise it will receive no attention.

*Daily Nursing.*

(220) Can you give me any idea of the fees a nurse engaged in daily nursing could expect among the middle classes and shopkeepers? I am thinking of starting nursing on these lines.—*Nurse Sophie.*

Miss C. J. Wood, Nurses' Hostel, 27, Percy Street, Tottenham Court Road, W.C., has carried on a system of "daily nursing" for some time Write to her on the subject.

*Male Nurses.*

(221) Will you tell me how and where to probation a male nurse? I have made enquiries at various institutions, but cannot get a satisfactory answer.—*A. W.*

There is no male nurse training school, unfortunately, in this country, though a few men are taken for training at the National Hospital for Paralysed and Epileptic, Queen Square, Bloomsbury. Have you written to the secretary, Mr. Burford Rawlings, for advice? You might also apply to Mr. F. Rouse, secretary to the Male Nurses' (Temperance) Co-operation, 10, Thayer Street, Manchester Square, W., or to the manager of the Hamilton Association for Providing Trained Male Nurses, 57, Park Street, Grosvenor Square, W.

*The Softening of Hard Water.*

(222) I am just taking possession of a new house at Winchester to which the town company supplies the water, which, I understand, comes from the chalk. Will you tell me the simplest way to soften this water for drinking purposes?—*J. S.*

When the hardness of water is of a temporary nature—that is to say, consists of calcic carbonate dissolved by aid of an excess of carbonic acid —it can be readily removed by Clark's process, which consists of the addition of sufficient quicklime to neutralise the free carbonic acid when practically the whole of the calcic carbonate, both that originally present and that produced by the combination of the quicklime, falls as a precipitate. Various forms of apparatus have been devised for the automatic performance of this process, and in some of them arrangements are made for filtering out the carbonate of lime which is produced, by which means the time is shortened.

*Training in Scotland.*

(223) Can you tell me where a nurse, who has already had two years' training, but who wishes to gain a certificate from a good general hospital, could take a two years' course in a good hospital in Scotland, earning a salary from the first.—*Scotch Nurse.*

You will not find it easy to get a two years' certificate from a good Scotch hospital. In most of them a three years' course is the rule. We should recommend you going in for the three years if you can give up the time, and applying to the matrons of the Edinburgh Royal Infirmary, the Aberdeen Royal Infirmary, the Glasgow Royal Infirmary, or any other of the well-known hospitals in Scotland. We believe at these institutions probationers are given a salary from the commencement of their training, or at least after the first year.

*Training.*

(224) Please tell me if any hospitals take probationers at 20 years of age, and what is the usual period of probation?—*A. B.*

You should get "How to Become a Nurse" (Scientific Press, 28 & 29, Southampton Street, Strand, London, W.C.). You will find there the terms of training at London and provincial hospitals, and the ages at which probationers are received. A good many children's hospitals admit probationers as young as 20. At general hospitals the usual age is more —23 to 25 as a rule. The time for training is usually three years.

*Male Nurses.*

(225) Is there any institution for training male nurses? Would a man of forty with a natural aptitude for nursing be suitable for training? —*J. W. T.*

See reply to "A. W." above.

*Midwifery Papers.*

(226) Please tell me if I can buy the back numbers of THE HOSPITAL containing the papers on "Midwifery," published last year? What would be the cost?—*Gratias.*

You can get the numbers you want from THE HOSPITAL Office, 28 & 29, Southampton Street, Strand, London, W.C. Write to the Manager about them.

*Royal National Pension Fund for Nurses.*

The Secretary of the Pension Fund has asked us to state that no reception of nurses at Marlborough House is contemplated for the present.

### Wants and Workers.

CAN any reader of THE HOSPITAL tell me of a home where an imbecile child of six years, subject to paralytic fits, could be admitted free of charge? The child's mother is a widow in poor circumstances with large family. Address Miss Lury, 62, Duke Street, Darlington.

